# Fibromyalgia: Associations Between Fat Infiltration, Physical Capacity, and Clinical Variables

**DOI:** 10.2147/JPR.S376590

**Published:** 2022-08-27

**Authors:** Björn Gerdle, Olof Dahlqvist Leinhard, Eva Lund, Ann Bengtsson, Peter Lundberg, Bijar Ghafouri, Mikael Fredrik Forsgren

**Affiliations:** 1 Pain and Rehabilitation Centre, and Department of Health, Medicine and Caring Sciences, Linköping University, Linköping, SE 581 83, Sweden; 2 Centre for Medical Image Science and Visualization (CMIV), Linköping, SE 581 83, Sweden; 3 Department of Radiation Physics, and Department of Health, Medicine and Caring Sciences, Linköping University, Linköping, SE 581 83, Sweden; 4 AMRA Medical AB, Linköping, Sweden

**Keywords:** body mass index, chronic pain, fibromyalgia, physical fitness, muscle, fat, magnetic resonance imaging, body composition

## Abstract

**Background:**

Obesity is a risk factor for the development of fibromyalgia (FM) and generally most studies report increased Body Mass Index (BMI) in FM. Obesity in FM is associated with a worse clinical presentation. FM patients have low physical conditioning and obesity further exacerbates these aspects. Hitherto studies of FM have focused upon a surrogate for overall measure of fat content, ie, BMI. This study is motivated by that ectopic fat and adipose tissues are rarely investigated in FM including their relationships to physical capacity variables. Moreover, their relationships to clinical variables including are not known. Aims were to 1) compare body composition between FM and healthy controls and 2) investigate if significant associations exist between body composition and physical capacity aspects and important clinical variables.

**Methods:**

FM patients (n = 32) and healthy controls (CON; n = 30) underwent a clinical examination that included pressure pain thresholds and physical tests. They completed a health questionnaire and participated in whole-body magnetic resonance imaging (MRI) to determine body composition aspects.

**Results:**

Abdominal adipose tissues, muscle fat, and BMI were significantly higher in FM, whereas muscle volumes of quadriceps were smaller. Physical capacity variables correlated negatively with body composition variables in FM. Both body composition and physical capacity variables were significant regressors of group belonging; the physical capacity variables alone showed stronger relationships with group membership. A mix of body composition variables and physical capacity variables were significant regressors of pain intensity and impact in FM. Body composition variables were the strongest regressors of blood pressures, which were increased in FM.

**Conclusion:**

Obesity has a negative influence on FM symptomatology and increases the risk for other serious conditions. Hence, obesity, dietary habits, and physical activity should be considered when developing clinical management plans for patients with FM.

## Introduction

Fibromyalgia (FM) has a prevalence of 1–4% in the population and has a higher prevalence in women.^1–3^ It is characterized by generalized widespread pain and hyperalgesia/allodynia.^4^ Symptoms and comorbidities such as fatigue, psychological distress, irritable bowel syndrome, and insomnia are frequent. FM diagnosis is either based on anamnestic reports and semi-objective examination of hyperalgesia and/or anamnestic reports.^4^,^5^

**Figure 1:**
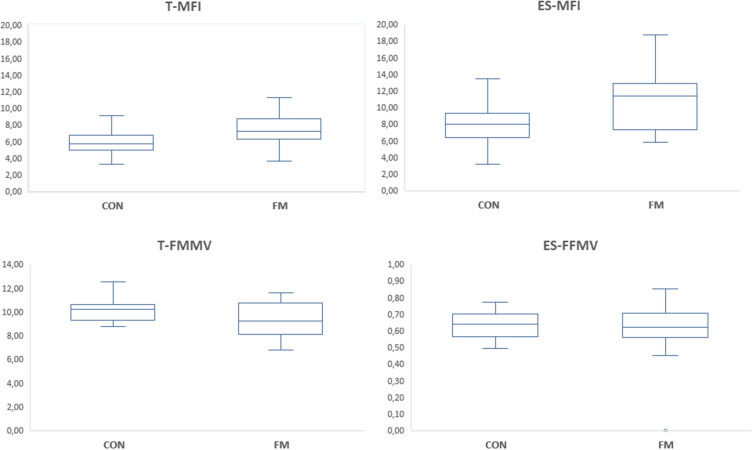
Boxplots of muscle fat infiltration (upper part) and fat-free muscle volume (lower part) in the thigh and erector spinae in healthy controls (CON) and fibromyalgia patients (FM). Note the different scales for T-FFMV and ES-FFMV.

**Abbreviations**: T-MFI, Thigh Muscle Fat Infiltration; ES-MFI, Erector Spinae Muscle Fat Infiltration; T-FFMV, Thigh Fat-free Muscle volume; ES-FFMV, Erector Spinae Fat-free Muscle volume.

The chronic pain field lacks mechanism-based diagnoses since the neurobiological alterations such as pathophysiological and molecular mechanisms are insufficiently known. The International Association for the Study of Pain (IASP) has defined nociplastic pain, such as nociceptive and neuropathic pain, as a pain mechanistic descriptor. FM is classified as a nociplastic pain condition,^6^ which includes characteristics such as increased anatomical spread of pain on the body and increased pain sensitivity. No definite pathophysiology for FM has been established and a pattern with alterations in different organs and tissues both at central and peripheral levels have been reported.^7–17^ Complicated interactions between peripheral and central processes are likely to be present in FM in conjunction with several psychosocial contributing factors.^18^

Obesity and increased fat content are associated with increased risk for conditions such as type 2 diabetes, hypertension, coronary heart disease, stroke, etc.^19–21^ Fibromyalgia is often accompanied by several comorbidities of which one of the most significant is obesity. Indeed, most studies report increased Body Mass Index (BMI) in FM;^22^,^23^ and a recent systematic review reported an overall obesity prevalence of 35.7% in FM.^24^ Also, obesity is a risk factor for the development of FM.^25^,^26^ Importantly, comorbid obesity further exacerbates the impairments associated with FM. Indeed, obesity in FM is associated with a worse clinical presentation, eg, in pain intensity, physical activity pattern, increased disability, and poor sleep, etc.^24^ High BMI in FM is associated with worse outcomes in pharmacotherapy and generally overweight and obesity are perceived as complex challenges in pain rehabilitation.^27^,^28^

Although BMI is an overall estimation of body mass including fat, muscles, and bones, it is used as a surrogate for overall measure of fat content. Therefore, a more detailed analysis of fat distribution in certain tissues may be warranted. Despite no significant group differences in BMI, significantly higher muscular fat infiltration (MFI) of quadriceps in FM than controls have been found.^29^ In whiplash associated disorders (WAD), increased MFI of cervical extensor musculature has been found.^30^

According to the deconditioning paradigm, physical inactivity and physical deconditioning can cause pain and contribute to intolerance of physical activities.^31^ Deconditioning may contribute to the development of FM.^32^ FM patients have reduced physical conditioning.^33–36^ Activity-induced pain is common and could help explain why FM patients avoid activities and exercise.^37^ Hence, we reported lower performance in FM on specific muscle function tests.^29^ Different physiological explanations for reduced strength in FM have been proposed – eg, alterations in muscle fiber, changes in neuromuscular control mechanisms, insufficient blood circulation, and alterations in energy and growth metabolism.^38^ The relationship between BMI and physical activity in FM is not clear.^39^ In a recent systematic review, only a weak association was found in FM.^24^ In this vicious cycle, several psychological factors play a role in patients with chronic pain and obesity. For example, with respect to chronic low back pain and obesity, recent evidence supported the role of kinesiophobia in contributing to pain intensity levels and disability,^40^,^41^ and more specifically, a recent study involving patients with obesity and FM corroborated the association between physical functioning and psychological factors such as pain catastrophizing and pain acceptance,^42^ highlighting that the cognitive and emotional response to pain stimuli affect behavior. The presence of several intertwining factors in the pain experience further complicates the understanding of the causal relationships.

Dietary habits in FM are insufficiently known even though some studies report significant changes in dietary patterns.^43^,^44^ Physical exercise is recommended in clinical guidelines for FM^33^ as both aerobic and muscle strengthening exercises are associated with reduced pain and improved well-being.^45^,^46^

To date, studies of FM have focused on the overall clinical estimate of normalized fat content – ie, BMI. However, the same BMI in two subjects can be associated with prominent differences in fat infiltration in specific tissues.^20^,^47^ Hence, ectopic fat and adipose tissues are rarely investigated in FM, including their relationships to physical capacity variables. Body composition profiling is a method that allows for assessing muscle volumes and fatty infiltration as well as visceral and abdominal subcutaneous adipose tissue volumes and liver fat infiltration using neck-to-knee MRI.^20^ Moreover, their relationships to clinical variables are not known.

Hence, this study has two main aims: a) to compare body composition of FM and healthy controls and b) to investigate whether significant associations exist between body composition and physical capacity aspects and important clinical variables (ie, pain intensity, psychological distress, disability, and blood pressure).

## Materials and Methods

### Subjects

This investigation of FM includes 32 female FM patients according to the 1990 American College of Rheumatology (ACR) criteria^4^ and 30 age-matched female controls (CON) between 22 and 56 years old. The CON group was recruited through advertisements in newspapers, and the FM group was recruited from former patients at the Pain and Rehabilitation Centre at the University Hospital in Linköping, Sweden. The number of subjects were determined using Power and sample size calculation (3.0.2)^48^ based on results both from microdialysis (concentration of lactate according to Rosendal et al^49^) and from ATP concentration obtained from phosphorus magnetic resonance spectroscopy^31^ of vastus lateralis.^29^ Both analyses indicated that 25 subjects in each group were necessary. Details about inclusion and exclusion criteria as well as clinical examination are given below.

The study was granted ethical clearance by Linköping University Ethics Committee (Dnr: 2016/239-31). All participants gave their written informed consent, and the study was performed in accordance with the Helsinki Declaration.

### Procedures

At the first visit, the subjects underwent a clinical examination. During the examination, pressure pain thresholds were registered, and physical tests were performed. In addition, the subjects completed a health questionnaire that covered aspects of pain, health, disability, demographic data, and psychological characteristics. At another visit, a whole-body magnetic resonance imaging (WB-MRI) scan was performed.

### Health Questionnaire

The questionnaire covered background data, pain aspects, psychological distress, disability, and health aspects; details including validity and the Swedish versions are reported elsewhere.^38^,^50^,^51^

#### Background Data

Age was registered.

#### Pain Aspects

Patients reported the duration of FM (years). Global pain intensity the previous seven days (NRS-7d) was reported using a numeric rating scale (0 = no pain and 10 = worst possible pain).

#### Psychological Distress

The Hospital Anxiety and Depression Scale (HADS) is used frequently in clinical practice and research.^52^,^53^ The total score (ie, the sum of the two subscales) was used to capture psychological distress (0–42).^51^ A lower score indicates fewer symptoms of distress.

#### Fibromyalgia Impact

The fibromyalgia impact questionnaire (FIQ) is comprised of ten subscales (each with a range of 0–100) of disabilities and symptoms.^54^ The mean of the ten subscales is calculated – higher score indicates higher impact (score range: 0–100).

#### Disability

The Pain Disability Index (PDI) measures the impact that pain has on a person’s ability to participate in essential life activities.^55^,^56^ PDI consists of seven items and every item is rated on a 10-point scale. The ratings of the items are summed, so the scores range between 0 and 70 (ie, high disability). The items assess perception of the specific impact of pain on disability that may preclude normal or desired performance of a wide range of functions such as sex, work, daily activities, family and social activities, and life support (sleeping, breathing, and eating).

#### Health Aspects

The European Quality of Life Instrument (EQ-5D), which captures a patient’s perceived state of health,^57^,^58^ consists of two parts. The first part is an index obtained from five dimensions. In this study, we used the second part – ie, the self-estimation of today’s health according to a 100-point thermometer-like scale (EQ5D-VAS) with defined end points (high values indicate better health and low values indicate worse health).

#### Physical Activity and Inactivity

Rating of Perceived Capacity (RPC) was used for self-assessment of perceived physical capacity.^59^,^60^ Using a scale from 1 to 20, the subjects reported, for example, what activity they could perform from a seated position (denoted 1) for at least 30 min, or, if athletes, what aerobic exercises they could perform (denoted 20) for at least 30 min. Seven other activities are denoted by a number.

Questions about physical inactivity and activity were taken from the Swedish National Board of Health and Welfare.^61^ One question was about the number of hours and minutes of sedentary time on an average day based on the last seven days (Physical inactivity (h)). Moreover, questions about the frequency (number of days per week) and average duration (min/day) of walking (Physical activity-2 min and Physical activity-2 days) as well as of moderately strenuous (Physical activity-3 min and Physical activity-3 days) and very strenuous physical activity (Physical activity-4 min and Physical activity-4 days) based on the previous seven days. The subjects were instructed to only consider physical activities that lasted for at least 10 min for these six variables.

### Clinical Examinations

Both FM and CON underwent a brief clinical examination of heart and lungs, which included recording diastolic and systolic blood pressure (mm Hg) after two minutes of rest in a horizontal position. The proportions with systolic blood pressure ≥130 mm Hg and/or diastolic ≥85 mm Hg were determined. In addition, their weight (kg) and height (m) were recorded. Body Mass Index (BMI, kg/m^2^) was calculated and classified according to the World Health Organization (WHO) criteria: <18.5 = underweight; 18.5–24.9 = normal range; 25.0–29.9 = overweight; 30.0–34.9 = obesity; and ≥35.0 = severe obesity. The clinical examination also ensured that the controls were healthy with respect to anamnesis for rheumatic diseases, neurological diseases, diabetes, cardio-vascular diseases, psychiatric diseases, and high alcohol consumption (ie, Alcohol Use Disorders Identification Test (AUDIT) >6 according to the recommendations for women).

The clinical examination of the patients ensured that they met the criteria for FM according to the 1990 American College of Rheumatology (ACR) criteria.^4^ The number of tender points were registered both in FM and in CON. The 1990 ACR criteria are based on anamnestic reports and semi-objective examinations of hyperalgesia/allodynia (tender points). Newer criteria (2010/2011 and 2016) based on anamnestic reports have been presented.^5^,^62^,^63^ As we wanted to compare our study with earlier studies and consensus is lacking concerning the 2010–2016 criteria,^64^ we chose the 1990 ACR criteria.

### Pressure Pain Thresholds

Pressure pain thresholds (PPT) were determined using a manual pressure algometer (Somedic AB, Sweden) mounted with a probe (contact area of 1 cm^2^) on the muscle belly (for details, see^65^,^66^). The erector spinae, tibialis anterior, and trapezius were investigated bilaterally. The pressure was increased by 30 kPa/s until the subject perceived pain, which the subject indicated by pushing a stop button, or until the maximum threshold of 600 kPa was reached. The PPT for each anatomical location was defined as the mean of two trials obtained with a minimum interval of 30s. We used the mean of the six anatomical locations (PPT-tot) as a global measure of pain sensitivity.

### Physical Tests

All subjects performed strength and endurance tests to determine hand function, aerobic fitness, and lower extremity muscle performance; the results of these have been recently reported.^67^ Hand function was determined by measuring grip force (N) using Grippit (AB Detektor, Gothenburg, Sweden). Peak value (Grip-max), average value (Grip-average), and 10s value (Grip-endurance) were recorded for 10s for both hands (for details, see our earlier study^29^). The test-retest precision has been shown to be generally high for these variables.^68^ Mean values of dominant and non-dominant sides are reported. To determine aerobic fitness, the subjects performed a submaximal cycle ergometer aerobic fitness test, which measures MaxVO_2._^69^ Lower extremity muscle performance was measured using the timed-stands test (TST) – ie, the number of times the subjects stand up and sit down from a standard chair for 30s.^70^,^71^

### Whole-Body Magnetic Resonance Imaging

Body composition was determined by quantitative analysis of magnetic resonance imaging (MRI). The participants were scanned in a Philips Ingenia 3T MRI scanner (Philips Healthcare, Netherlands) using a 6-min dual-echo Dixon protocol, providing water and fat separated volumetric data covering a region from the neck to the knees. In addition, a dedicated mDixon Quant protocol was used to quantify liver fat.^72^ The body composition profiling was performed using AMRA® Researcher (AMRA Medical AB, Linköping, Sweden).^20^ The analysis consisted of the following steps: 1) automatic image calibration, 2) automatic labelling and registration of fat and muscle regions to the acquired image volumes, 3) quality control of anatomical regions and MR data performed by trained analysis engineers at AMRA Medical, and 4) quantification of fat and muscle volumes based on the calibrated images.^72–76^ The measurements included the volumes of visceral adipose tissue (VAT (L)), and abdominal subcutaneous adipose tissue (ASAT (L)), thigh fat-free muscle volume (T-FFMV (L)), fat-free muscle volume of spinal erectors (ES-FFMV (L)), the relative fat infiltration of these muscle groups (T-MFI and ES-MFI; (%)), as well as liver proton density fat-fraction (Liver fat (%)). The spinal erector group (‘spinal erectors’) measurement consisted of the following muscles iliocostalis, longissimus, spinalis, and transversospinales due to the inability to exclude this small muscle group from the rest given the spatial resolution of the image data. The spinal erectors were measured in a region limited by the top of first lumbar vertebrae (L1) and the bottom of the fifth lumbar vertebrae (L5).

For each subject, a matched virtual control group (VCG) was created to calculate a personalized muscle volume z-score based on T-FFMV (labelled T-MVZ). The muscle volume z-score measures how many standard deviations each subject deviates from the mean muscle volume of their matched group, which represents the same gender and similar body size. The reference population from which the VCG was drawn consisted of 10016 subjects aged 44 to 76 from the UK Biobank imaging sub-study.^77^ This research was conducted using the UK Biobank resource, project ID 6569. The study was approved by the Northwest Multicentre Research Ethics Committee, UK. Written informed consent was obtained before study entry. Below, BMI and the variables obtained from the whole-body MRI are labelled body composition variables.

### Statistics

The statistics were performed using the statistical packages IBM SPSS Statistics (version 27.0; IBM Corporation, Route 100 Somers, New York, USA) and SIMCA-P+ (version 17.0; Sartorius Stedim Biotech, Umeå, Sweden). A P-value <0.05 was considered statistically significant. Text and tables report the mean value ± one standard deviation (± 1 SD) of continuous variables and percentages (%) for categorical variables. To compare groups, we used Student’s *t*-test for un-paired observations and Chi squared test for proportions.

Previous studies have discussed the necessity of using advanced multivariate analyses (MVDA) when accounting for system-wide aspects, including missing data and multicollinearity problems.^50^,^78^ Using SIMCA-P+, we applied advanced Principal Component Analysis (PCA) to determine multivariate outliers and multivariate correlation patterns and Orthogonal Partial Least Square Regressions (OPLS) to determine multivariate associations. SIMCA-P+ uses the Non-linear Iterative Partial Least Squares (NIPALS) algorithm to handle missing data: max 50% missing data for variables/scales and max 50% missing data for subjects. PCA extracts and displays systematic variation in the data matrix (ie, a kind of multivariate correlation analysis). A cross validation technique was used to identify nontrivial components (p). Variables loading on the same component p were correlated, and variables with high loadings but opposing signs were negatively correlated. Variables with high absolute loadings were considered significant. Per definition, the obtained components are not correlated and are arranged in decreasing order with respect to explained variation. R^2^ describes the goodness of fit – the fraction of sum of squares of all the variables explained by a principal component.^79^ Q^2^ describes the goodness of prediction – the fraction of the total variation of the variables that can be predicted using principal component cross validation methods.^79^

OPLS-discriminant analysis (OPLS-DA) was made for multivariate group analysis (CON or FM). OPLS was used for the multivariate regression analyses of the clinical variables both in all subjects taken together and in the two groups separately. The variable influence on projection (VIP) indicates the relevance of each X–variable pooled over all dimensions and Y-variables – the group of variables that best explains Y.^79^ VIP > 1.0 (or VIPpred if more than one component was identified) was considered significant if VIP had 95% jack-knife uncertainty confidence interval non-equal to zero. P(corr) was used to note the direction of the relationship (positive or negative) – ie, the loading of each variable was scaled as a correlation coefficient and therefore standardized the range from −1 to +1.^78^ P(corr) is stable during iterative variable selection and comparable between models. An absolute p(corr) of ≥0.50 was considered statistically significant.^78^ Thus, a variable/regressor was considered statistically significant when VIP > 1.0 and absolute p(corr) ≥0.50. A regression model will be obtained, including one or several components (the first is always the predictive component), when certain predefined criteria are fulfilled. The validity of the model is estimated using cross validation. Hence, for each regression, we report R^2^, Q^2^, and the P-value of a cross-validated analysis of variance (CV-ANOVA).

## Results

### Clinical Presentation

Most of the clinical variables, except diastolic blood pressure and age, differed between the two groups of subjects (Table 1). Hence, compared to the controls, patients reported higher systolic pressure, moderate to high pain intensities, together with significantly higher psychological distress, higher disability, and lower health. Systolic blood pressure was ≥130 mm Hg for 31% of FM and 7% of CON (p = 0.023), and diastolic blood pressure was ≥85 mm Hg for 22% of FM and 10% of CON (p = 0.304). Moreover, FM had lower PPT-tot and more tender points than CON.

**Table 1:** Data Obtained from Questionnaires and at the Clinical Examinations in Controls (CON) and in Fibromyalgia Patients (FM) (Mean and SD). Furthest to the Right are the Results of Group Comparisons (P-value and Effect Size (ES) According to Cohen’s d)

Group	CON	N=30	FM	N=32	Statistics	ES
Variables	Mean	SD	Mean	SD	P-value	Cohen’s d
Age	42.80	9.88	40.00	11.23	0.309	0.21
Blood pressure systolic (mm Hg)	113.47	8.76	120.47	11.69	0.011	−0.73
Blood pressure diastolic (mm Hg)	75.83	8.25	80.10	8.57	0.054	−0.57
PPT-tot	385.01	111.53	138.24	86.76	<0.001	2.54
Nos. tender points	0.33	0.88	16.47	2.73	<0.001	−7.95
FM duration (years)	NA	NA	5.63	5.76	NA	NA
NRS-7d	0.00	0.00	6.54	1.69	<0.001	−5.61
HADS	4.23	3.65	13.32	6.11	<0.001	−1.89
FIQ	NA	NA	59.78	16.76	NA	NA
PDI	8.48	5.01	37.25	10.88	<0.001	−3.44
EQ5D-VAS	86.90	7.57	51.86	18.84	<0.001	2.48

**Notes**: Adapted from: Gerdle B, Ghafouri B, Lund E, et al. Evidence of Mitochondrial Dysfunction in Fibromyalgia: Deviating Muscle Energy Metabolism Detected Using Microdialysis and Magnetic Resonance. *J Clin Med*. 2020;9(11):3527.^67^ Copyright © 2020 by the authors. Licensee MDPI, Basel, Switzerland. Creative Commons Attribution (CC BY) license (http://creativecommons.org/licenses/by/4.0/).

**Abbreviations**: CON, controls; FM, fibromyalgia; N, number of subjects; SD, standard deviation; ES, effect size; PPT-tot, Pressure Pain Threshold, mean of six sites; NRS-7d, global pain intensity previous 7 days using a numeric rating scale (NRS); HADS, The Hospital Anxiety and Depression Scale; FIQ, Fibromyalgia impact questionnaire; PDI, Pain Disability Index; EQ5D-VAS, Perceived Health according to European Quality of life (EQ) instrument; NA, Not Applicable.

### Body Composition Variables

BMI was significantly higher in FM than in CON (Table 2). The proportions underweight/normal weight, overweight, and obesity were 28%, 28%, and 44% in FM and 70%, 23%, and 7% in CON, respectively (Chi squared = 14.0, df = 2, P < 0.001). Hence, the proportion obesity was >6 times higher in FM.

**Table 2:** Body Composition Variables in Controls (CON) and in Fibromyalgia Patients (FM) (Mean and SD). Furthest to the Right are the Results of Group Comparisons (P-value and Effect Size (ES) According to Cohen’s d)

Group	CON	N=30	FM	N=32	Statistics	ES
Variables	Mean	SD	Mean	SD	P-value	Cohen’s d
BMI (kg/m^2^)	23.98	3.07	29.15	6.07	<0.001	−1.12
VAT [L]	1.272	0.803	3.130	2.017	<0.001	−1.19
ASAT [L]	6.420	2.957	11.101	5.494	<0.001	−1.05
Liver Fat [%]	1.8	0.9	5.5	7.2	0.010	−0.69
T-MFI [%]	5.9	1.2	7.5	1.9	<0.001	−1.03
ES-MFI [%]	8.0	2.3	10.4	3.6	0.003	−0.79
T-FFMV [L]	10.111	0.945	9.336	1.503	0.021	0.61
ES-FFMV [L]	0.634	0.082	0.623	0.153	0.734	0.09
T-MVZ [SDs]	1.514	0.766	0.295	1.292	<0.001	1.13

**Notes**: BMI data have been published elsewhere: adapted from: Gerdle B, Ghafouri B, Lund E, et al. Evidence of Mitochondrial Dysfunction in Fibromyalgia: Deviating Muscle Energy Metabolism Detected Using Microdialysis and Magnetic Resonance. *J Clin Med*. 2020;9(11):3527.^67^ Copyright © 2020 by the authors. Licensee MDPI, Basel, Switzerland. Creative Commons Attribution (CC BY) license (http://creativecommons.org/licenses/by/4.0/).

**Abbreviations**: CON, controls; FM, fibromyalgia; N, number of subjects; SD, standard deviation; BMI, Body Mass Index; VAT, Visceral Adipose Tissue volume; ASAT, Abdominal Subcutaneous Adipose Tissue volume; T-MFI, Thigh Muscle Fat Infiltration; ES-MFI, Erector Spinae Muscle Fat Infiltration; T-FFMV, Thigh Fat-free Muscle volume; ES-FFMV, Erector Spinae Fat-free Muscle volume; T-MVZ, muscle volume z-score, ie, deviations from expected T-FFMV.

The volumes of visceral and abdominal subcutaneous adipose tissue (VAT and ASAT) and percentage of liver fat were significantly higher in FM than CON (Table 2). The fat infiltration in muscles – ie, in thigh (T-MFI) and in erector spinae (ES-MFI) – were significantly higher in FM than in the CON (Table 2; Figure 1). Moreover, the total fat free muscle volume of the thighs (T-FFMV), but not of erector spinae (ES-FFMV), was significantly lower in FM than CON (Table 3; Figure 1). Also, T-MVZ differed significantly between the two groups.

**Table 3:** Physical Capacity Variables Obtained from Self-Reports and from Physical Tests (Mean and SD) in Controls (CON) and in Patients with FM. Furthest to the Right are the Results of Group Comparisons (P-value and Effect Size (ES) According to Cohen’s d)

Group	CON	N=30	FM	N=32	Statistics	Effect Size
Variables	Mean	SD	Mean	SD	P-value	Cohen’s d
***Self-reported***						
Physical inactivity (h)	7.64	6.01	11.14	15.77	0.277	−0.29
Physical activity-2 Days	6.50	1.46	5.30	2.38	0.022	0.61
Physical activity-2 min	144.93	183.41	191.30	197.33	0.366	−0.24
Physical activity-3 Days	3.87	2.46	2.13	2.11	0.005	0.76
Physical activity-3 min	72.24	70.49	63.07	86.97	0.663	0.12
Physical activity-4 Days	2.63	2.13	1.27	2.07	0.014	0.65
Physical activity-4 min	40.76	31.17	28.66	64.39	0.366	0.24
RPC	10.28	2.17	6.27	2.94	<0.001	1.55
***Physical tests***						
MaxVO_2_ (mL/kg/min)	2.66	0.50	2.17	0.53	0.001	0.94
Grip-max (N)	315.44	47.90	240.13	64.96	<0.001	1.31
Grip-average (N)	235.09	44.21	159.87	53.05	<0.001	1.53
Grip-endurance (N)	236.17	128.12	136.15	47.54	<0.001	1.06
TST	17.90	3.04	13.39	3.25	<0.001	1.43

**Notes**: The results of the physical tests have been published elsewhere: adapted from: Gerdle B, Ghafouri B, Lund E, et al. Evidence of Mitochondrial Dysfunction in Fibromyalgia: Deviating Muscle Energy Metabolism Detected Using Microdialysis and Magnetic Resonance. *J Clin Med*. 2020;9(11):3527.^67^ Copyright © 2020 by the authors. Licensee MDPI, Basel, Switzerland. Creative Commons Attribution (CC BY) license (http://creativecommons.org/licenses/by/4.0/).

**Abbreviations**: CON, controls; FM, fibromyalgia; N, number of subjects; SD, standard deviation; for explanation of self-reported physical inactivity and activity variables see methods; RPC, Rating of Perceived Capacity; MaxVO_2_, Aerobic fitness test; Grip, Grip force; Grip-max, Peak value of grip force; Grip-average, average value of grip force; Grip-endurance, 10-second value of grip force; TST, timed-stands test.

The relationship between BMI and the other registered body composition variables were investigated. Hence, a highly significant OPLS regression of BMI was found in all subjects together (R^2^=0.88, Q^2^=0.84, CV-ANOVA P-value: 5.96E-20). Hence, ASAT, VAT, and T-MFI were the significant variables and correlated positively with BMI. Also, FM and in CON each had highly significant regressions that identified ASAT, VAT, and T-MFI as significantly associated with BMI (FM: R^2^ = 0.88, Q^2^ = 0.82, CV-ANOVA P-value: 1.04E-09; CON: R^2^ = 0.69, Q^2^ = 0.61, CV-ANOVA P-value: 4.98E-06). In CON, ES-MFI was also significant (third in importance before T-MFI).

We then regressed (OPLS-DA) group membership using the body composition variables (cf. Table 2) as regressors (R^2^ = 0.38, Q^2^ = 0.35, CV-ANOVA P-value = 3.80e-06). The following four variables – in descending order of importance – were significant and higher in FM than in CON: VAT (VIP = 1.27, p(corr) = 0.88); T-MFI (VIP = 1.25, p(corr) = 0.87); ASAT (VIP = 1.22, p(corr) = 0.85); and BMI (VIP = 1.21, p(corr) = 0.84).

### Physical Capacity

According to most of the self-reports (ie, the three physical activity-days variables and RPC), FM reported significantly lower capacity (Table 3). As reported elsewhere, this FM cohort had lower physical capacity than CON according to the physical tests (Table 3).^67^ Hence, FM had lower self-reported and objective physical capacity in most investigated aspects.

When regressing group membership using both the self-reported and the physical test variables (Table 3) as regressors, a significant model was obtained (R^2^ = 0.52, Q^2^ = 0.48, CV-ANOVA P-value = 9.49e-09). The following variables were significant and correlated negatively with the FM group membership: Grip-average (VIP = 1.37, p(corr) = −0.84); Grip-max (VIP = 1.33, p(corr)= −0.82), RPC (VIP = 1.28, p(corr) = −0.79); TST (VIP = 1.21, p(corr) = −0.75); Physical activity-4 min (VIP = 1.12, p(corr) = −0.68); MaxVO_2_ (VIP = 1.08, p(corr) = −0.65); and Physical activity-3 days (VIP = 1.06, p(corr) = −0.65). Hence, a mix of self-reported variables and physical tests influenced group differentiation.

### Relationships Between Physical Capacity and Body Composition Variables

The interrelationships between the physical capacity variables and body composition variables were analyzed using PCAs in all subjects together (R^2^cummulative = 0.52) and in FM (R^2^cummulative = 0.49) and in CON (R^2^cummulative = 0.41) using the two first most important components (see Table S1 in Supplementary text #1). These three analyses showed that some of the physical capacity variables (both subjective and objective) correlated negatively with several of the body composition variables even though the variables differed somewhat across the analyses. Hence, in FM VAT and liver fat correlated negatively and strongest with the three grip-variables and TST as well as with RPC and MaxVO_2_ (Table S1 in Supplementary text #1).

### Regression of Group Membership Using Body Composition Variables and Physical Capacity Variables

The OPLS-DA regression of group (FM or CON) using body composition variables and physical capacity variables (Tables 2 and 3) as regressors was highly significant (R^2^ = 0.53, Q^2^ = 0.48, CV-ANOVA P-value = 4.26e-09) (Table 4). A mix of variables were significant and contributed to differentiate FM and CON – ie, physical tests (Grip-average, Grip-max, TST and MaxVo2), self-reported physical capacity (RPC), and body composition (VAT, T-MFI, T-MVZ, BMI, ASAT, and Liver fat) (Table 4).

**Table 4:** OPLS-DA Regression of Group Membership (CON Denoted as 0 and FM Denoted as 1) Using Fat Infiltration Variables (Cf. Table 2) and Physical Capacity Variables (Self-Reports and Physical Tests; Cf. Table 3) as Regressors. Significant Variables in Bold Type

Variables	VIP	p(corr)
**Grip-average**	**1.41**	−**0.80**
**VAT**	**1.35**	**0.77**
**RPC**	**1.34**	−**0.78**
**Grip-max**	**1.34**	−**0.77**
**TST**	**1.32**	−**0.76**
**T-MFI**	**1.29**	**0.74**
**T-MVZ**	**1.27**	−**0.73**
**BMI**	**1.20**	**0.68**
**ASAT**	**1.18**	**0.68**
**Liver fat**	**1.13**	**0.65**
**MaxVO_2_**	**1.06**	−**0.60**
ES-MFI	0.94	0.54
Physical activity-4 Days	0.94	−0.54
Grip-endurance	0.89	−0.51
Physical activity-3 Days	0.84	−0.48
T-FFMV	0.65	−0.37
Physical activity-2 Days	0.60	−0.34
Physical activity-4 min	0.38	−0.22
Physical activity-2 min	0.22	0.12
ES-FFMV	0.14	−0.08
Physical activity-3 min	0.14	−0.08
Physical inactivity	0.13	0.08
R^2^	0.53	
Q^2^	0.48	
P-value	4.26e-09	

**Notes**: VIP and p(corr) are reported for each regressor (ie, the loading of each variable scaled as a correlation coefficient and therefore standardizing the range from −1 to +1). The sign of p(corr) indicates the direction of the correlation with the dependent variable (+ = positive correlation, ie, higher value in FM; − = negative correlation, ie, higher in CON). The three bottom rows report R^2^, Q^2^, and P-value of the CV-ANOVA. For explanation of self-reported physical activity variables, see methods.

**Abbreviations**: RPC, Rating of Perceived Capacity; MaxVO_2_, Aerobic fitness test; Grip, Grip force; Grip-max, Peak value of grip force; Grip-average, average value of grip force; Grip-endurance, 10-second value of grip force; TST, timed-stands test; BMI, Body Mass Index; VAT, Visceral Adipose Tissue volume; ASAT, Abdominal Subcutaneous Adipose Tissue volume; T-MFI, Thigh Muscle Fat Infiltration; ES-MFI, Erector Spinae Muscle Fat Infiltration; T-FFMV, Thigh Fat-free Muscle volume; ES-FFMV, Erector Spinae Fat-free Muscle volume; T-MVZ, muscle volume z-score, ie, deviation from expected T-FFMV.

### Clinical Variables – Regression Analyses

#### Regression of Pain Intensity in FM

When pain intensity in FM was regressed using the physical capacity variables and the body composition variables, a mix of variables were significantly associated with NRS-7d (R^2^ = 0.45, Q^2^ = 0.32, CV ANOVA P-value = 0.0055) (Table 5). Hence, VAT, Liver fat, BMI, T-MFI, and ASAT were positively associated with pain intensity, and TST, the three grip variables, and RPC showed negative associations. VAT, liver fat, and TST had the strongest associations with pain intensity (Table 5).

**Table 5:** OPLS Regression of Pain Intensity (NRS-7d) in Fibromyalgia Patients (FM) Using the Fat Infiltration Variables (Cf. Table 2) and the Physical Capacity Variables (Self-Reports and Physical Tests; Cf. Table 3) as Regressors (x–Variables). Variables in Bold are Significant

Variables	VIPpred	p(corr)
**VAT**	**1.52**	**0.77**
**Liver Fat**	**1.46**	**0.74**
**TST**	**1.39**	−**0.70**
**Grip-average**	**1.38**	−**0.70**
**Grip-endurance**	**1.33**	−**0.68**
**BMI**	**1.33**	**0.67**
**T-MFI**	**1.31**	**0.66**
**Grip-max**	**1.27**	−**0.64**
**RPC**	**1.23**	−**0.62**
**ASAT**	**1.21**	**0.61**
MaxVO_2_	0.91	−0.48
T-MVZ	0.90	−0.46
Physical activity-4 Days	0.84	−0.43
Physical activity-3 Days	0.71	−0.36
Physical activity-4 min	0.62	−0.32
ES-MFI	0.62	0.31
Physical activity-3 min	0.33	−0.17
Physical activity-2 Days	0.25	−0.13
Physical inactivity	0.21	−0.10
ES-FFMV	0.18	0.09
Physical activity-2 min	0.08	−0.04
T-FFMV	0.03	−0.02
R^2^	0.45	
Q^2^	0.32	
P-value	0.0055	

**Notes**: VIPpred and p(corr) are reported for each regressor (ie, the loading of each variable scaled as a correlation coefficient and therefore standardizing the range from −1 to +1). The sign of p(corr) indicates the direction of the correlation with the dependent variable (+ = positive correlation; − = negative correlation). The three bottom rows report R^2^, Q^2^, and P-value of the CV-ANOVA. For explanation of self-reported physical activity variables see methods.

**Abbreviations**: RPC, Rating of Perceived Capacity; MaxVO_2_, Aerobic fitness test; Grip, Grip force; Grip-max, Peak value of grip force; Grip-average, average value of grip force; Grip-endurance, 10-second value of grip force; TST, timed-stands test; BMI, Body Mass Index; VAT, Visceral Adipose Tissue volume; ASAT, Abdominal Subcutaneous Adipose Tissue volume; T-MFI, Thigh Muscle Fat Infiltration; ES-MFI, Erector Spinae Muscle Fat Infiltration; T-FFMV, Thigh Fat-free Muscle volume; ES-FFMV, Erector Spinae Fat-free Muscle volume; T-MVZ, muscle volume z-score, ie, deviation from expected T-FFMV.

A significant OPLS regression of NRS-7d was also obtained using only the physical capacity variables (R^2^ = 0.40, Q^2^ = 0.29, CV ANOVA P-value = 0.0083); the three Grip variables followed by TST, and RPC were significant regressors. No significant regression was obtained using only the fat infiltration variables as regressors (X–variables) of NRS-7d.

#### Regression of FIQ in FM

A significant regression was obtained for FIQ in FM using the physical capacity variables and the body composition variables (R^2^ = 0.45, Q^2^ = 0.33, CV ANOVA P-value = 0.0046) (Table 6). Hence, physical capacity variables (three grip variables, TST, RPC, and MaxVO_2_) showed significant negative associations with FIQ. VAT, liver fat, T-MFI, BMI, ASAT, and T-MVZ showed significant positive associations. Two grip variables, TST, and VAT showed the strongest associations with FIQ (Table 6).

**Table 6:** OPLS Regression of FIQ in Fibromyalgia Patients (FM) Using the Fat Infiltration Variables (Cf. Table 2) and the Physical Capacity Variables (Self-Reports and Physical Tests; Cf. Table 3) as Regressors (x–Variables). Variables in Bold Type are Significant

Variables	VIPpred	p(corr)
**Grip-average**	**1.48**	−**0.75**
**TST**	**1.45**	−**0.74**
**Grip-endurance**	**1.43**	−**0.73**
**VAT**	**1.42**	**0.72**
**Liver Fat**	**1.38**	**0.70**
**Grip-max**	**1.37**	−**0.70**
**T-MFI**	**1.27**	**0.65**
**BMI**	**1.20**	**0.61**
**RPC**	**1.15**	−**0.58**
**ASAT**	**1.12**	**0.57**
**MaxVO_2_**	**1.04**	−**0.55**
**T-MVZ**	**1.03**	−**0.52**
Physical activity-4 Days	0.81	−0.41
Physical activity-3 Days	0.71	−0.36
Physical activity-4 min	0.57	−0.30
ES-MFI	0.56	0.28
Physical activity-2 Days	0.33	−0.17
Physical activity-3 min	0.28	−0.15
T-FFMV	0.23	−0.12
Physical activity-2 min	0.16	−0.09
Physical inactivity	0.16	−0.07
ES-FFMV	0.05	0.03
R^2^	0.45	
Q^2^	0.33	
P-value	0.0046	

**Notes**: VIPpred and p(corr) are reported for each regressor (ie, the loading of each variable scaled as a correlation coefficient and therefore standardizing the range from −1 to +1). The sign of p(corr) indicates the direction of the correlation with the dependent variable (+ = positive correlation; − = negative correlation). The three bottom rows report R^2^, Q^2^, and P-value of the CV-ANOVA. For explanation of self-reported physical activity variables see methods.

**Abbreviations**: RPC, Rating of Perceived Capacity; MaxVO_2_, Aerobic fitness test; Grip, Grip force; Grip-max, Peak value of grip force; Grip-average, average value of grip force; Grip-endurance, 10-second value of grip force; TST, timed-stands test; BMI, Body Mass Index; VAT, Visceral Adipose Tissue volume; ASAT, Abdominal Subcutaneous Adipose Tissue volume; T-MFI, Thigh Muscle Fat Infiltration; ES-MFI, Erector Spinae Muscle Fat Infiltration; T-FFMV, Thigh Fat-free Muscle volume; ES-FFMV, Erector Spinae Fat-free Muscle volume; T-MVZ, muscle volumes z-score, ie, deviations from expected T-FFMV.

A significant OPLS regression was also obtained when only using the physical capacity variables as regressors (R^2^ = 0.39, Q^2^ = 0.24, CV ANOVA P-value = 0.027); the three grip variables, TST, and MaxVo2, in that order, were significant regressors. No significant regression was obtained using the body composition variables alone as regressors (X–variables).

#### Regression of Blood Pressures in FM

In FM (but not in CON), it was possible to significantly regress blood pressures. Body composition variables were the most important variables for both diastolic and systolic blood pressures in FM. When we also included clinical variables (ie, NRS-7d, HADS, PDI, tender points, FIQ, and PPT-tot) in the analysis, this pattern remained and the following variables were significant (R^2^ = 0.47, Q^2^ = 0.32, CV-ANOVA P-value = 0.004, one predictive component): VAT (VIP = 1.78, p(corr) = 0.86); BMI (VIP = 1.65, p(corr) = 0.79); ASAT (VIP = 1.62, p(corr) = 0.78); T-MFI (VIP = 1.50, p(corr) = 0.72); FIQ (VIP = 1.38, p(corr) = 0.68); NRS-7d (VIP = 1.37, p(corr) = 0.68); Grip average (VIP = 1.23, p(corr) = −0.59); liver fat (VIP = 1.18, p(corr) = 0.57); Grip-endur (VIP = 1.17, p(corr) = −0.56); TST (VIP = 1.11, p(corr) = −0.52); Grip-max (VIP = 1.09, p(corr) = −0.52); PDI (VIP = 1.06, p(corr) = 0.53); and ES-MFI (VIP = 1.01, p(corr) = 0.49).

Diastolic blood pressure resulted in a very similar pattern of important variables with the four most important the same and in the same order (data not shown) (R^2^ = 0.55, Q^2^ = 0.33, CV-ANOVA P-value = 0.003, and one predictive component).

#### Regressions of PPT-TOT, HADS, PDI, and EQ5D-VAS

A significant regression of PPT-tot was obtained for all subjects taken together but not separately in the two groups using the physical capacity variables and the body composition variables (data not shown). However, due to the prominent group differences in PPT, the results were very similar to the regression of group. Similar situations existed when regressing HADS, PDI, and EQ5D-VAS (data not shown).

## Discussion

The following major results were obtained: 1) The adipose-tissue-related body composition variables, ie, VAT, ASAT, liver fat, T-MFI, ES-MFI, and BMI were significantly higher in FM. The FM group also had significantly smaller thigh muscles (T-FFMV and T-MVZ), but the spinal erector volume was not significantly different. 2) FM self-reported lower physical capacity, which agrees with results for objective physical tests reported earlier for this cohort.^67^ 3) Both body composition variables and physical capacity variables were significantly associated with group belonging; the physical capacity variables showed stronger relationships with group membership than the body composition variables. 4) Physical capacity variables and several of the body composition variables intercorrelated negatively in FM. 5) Both pain intensity and FIQ were significantly associated with a blend of body composition variables and physical capacity variables. The physical capacity variables alone but not body composition variables could significantly regress both variables. 6) In FM, blood pressures were significantly associated with the body composition variables.

It is established that FM is associated with increased BMI and obesity,^24^ which also was found in the present study, and the prevalence of obesity was increased >6 times. BMI is an overall measure of body mass including fat, muscles, bones, etc. and is used as a surrogate for overall measure of fat content. In certain situations, BMI may not accurately reflect body fat content and other clinical measures have been proposed.^47^,^80^,^81^ Here, fat infiltration in two muscles and liver, viscera, and abdomen adipose tissue volumes were obtained. VAT, ASAT, and T-MFI were significantly associated with BMI; the regression explained 88% of the variation in BMI. Moreover, VAT was the most important fat content variable for group membership, and in FM for pain intensity, FIQ and blood pressures. VAT together with liver fat also correlated negatively with physical capacity variables in FM. Hence, it was a more important factor than BMI in these aspects. BMI, liver fat, ASAT, and T-MFI were also significant factors, but their relative importance varied across regressions. As recently summarized, increased VAT and ASAT are associated with increased risks for type 2 diabetes, cardiovascular diseases, sleep apnea, chronic obstructive pulmonary disease, stroke, and brain conditions including cancer.^19^ Linge et al reported that higher VAT, MFI, and liver fat but not ASAT were associated with coronary heart disease and type 2 diabetes,^20^ which are also prospectively verified for VAT and liver fat.^21^ In obese subjects, VAT and ASAT secrete, eg, inflammatory and immune compounds, and micro-RNAs, which can exert functional and pathophysiological alterations in various tissues.^19^

In another FM cohort, we reported increased MFI in the quadriceps muscle despite no significant group differences in BMI.^29^ The higher MFI is confirmed here, and we found increased MFI of the ES muscle group in FM. Hence, MFI in FM was significantly higher both in weightbearing muscles (thighs) as well as the spinal muscles.

Significantly higher fat infiltration in muscles within painful areas has been found in chronic low back pain^82–84^ and in chronic WAD^30^,^85–88^ compared to controls without significantly different BMI.^84^,^85^,^87^,^89^ Some studies indicate that fat infiltration in chronic WAD is local to the neck and not generalized^89^,^90^ and therefore is a direct or indirect consequence of acute trauma and/or pain. In chronic WAD, increased fat infiltration has been associated with clinical severity and poor recovery.^85^,^91^ Different explanations exist for the increased muscle fat infiltration in chronic WAD,^87^ and the influences of eg, age, sex, BMI, physical activity and stress, are insufficiently known.^85^ Local muscle fat infiltration may be associated with increased cytokine levels.^92^ Hence, a relationship between fat accumulation and inflammatory dysregulation was indicated even though a causal direction could not be established.^92^

In contrast to these local/regional chronic pain conditions, FM had higher BMI, increased fat infiltration in muscles and liver, and higher visceral and abdominal fat volumes. These findings could be the result of deconditioning in FM.^33–35^ We recently reported lower capacity according to physical tests for the present and another FM cohort.^29^,^67^ Most FM studies have used either self-reports or objective tests to assess physical activity levels.^42^ In the present study, several of the subjective reports agreed with the objective physical tests. For FM, longer sedentary time, sleep duration, and lower physical activity levels are independently associated with greater adiposity.^93^ The widespread pain in FM may be a barrier for physical activity and therefore deconditioning, increasing fat infiltration in a vicious circle. The present results’ significant negative associations in both groups between fat content and physical capacity variables support the idea that deconditioning is an important explanation for the increased fat infiltration even though this is a cross-sectional study. The lower FFMV in the thigh – a large weight-bearing muscle group important for mobility and physical activity – partly supports this interpretation.

Possibly, muscle fat infiltration in FM to some extent could be related to generalized pathophysiological processes in muscles and blood. For example, the present FM cohort showed signs of muscle mitochondrial dysfunction.^67^ In chronic widespread pain cohorts (mainly FM), have been found increased muscle interstitial metabolite concentrations,^94–96^ decreased phosphocreatine (PCr) and Adenosine Tri-Phosphate (ATP) muscle levels,^29^,^67^ and alterations in muscle lipid mediators.^97^,^98^ In blood, there are significant alterations in protein patterns related to immunity and inflammation^99–102^ and in lipid mediators.^103–106^ Hence, local/regional as in WAD and LBP or generalized peripheral nociceptive processes may contribute to increased fat infiltration.

Our study agrees with previous reports that obesity is associated with a worse clinical presentation (eg, pain intensity, disability, and sleep) in FM.^22^,^24^,^107^ However, these correlations have been weak in many studies.^108^ Both impact and pain intensity increased with increased fat infiltration according to the regressions (Tables 5 and 6). Segura-Jimenez et al reported significant associations between impact and total and central body fat.^108^ They suggested that the physical fitness may explain the association between obesity and FM symptoms.^108^ In agreement with those results were found that a mix of fat infiltration and volume variables and physical capacity variables were significantly associated with pain intensity and FIQ in FM. The combination of increased MFI with low FFMV has been shown to be strongly linked to poor activities of daily life, hospitalization, and all-cause mortality in the general population.^77^,^109^ Hence, signs of deconditioning together with more fat infiltration and volumes were associated with a worse clinical presentation. Our analysis shows, as in other studies, that physical activity performance is more strongly associated with pain intensity and FIQ than fat-related variables.

Obesity is a risk factor for hypertension.^81^ FM patients had significantly higher systolic blood pressure and a similar non-significant trend for diastolic pressure. An increased prevalence of hypertension is found in subjects with chronic pain^110^,^111^ and is more prevalent in widespread pain than in localized pain.^111^ This study found a significantly higher proportion of FM with a systolic blood pressure ≥130 mm Hg. Most studies report high prevalence of Metabolic syndrome (MetS) in FM,^112–114^ which is associated with increased risk for cardiovascular disease, diabetes mellitus, and all-cause mortality.^113^,^115^ The increased VAT, ASAT, liver fat, and BMI together with nearly a third of the patients displaying systolic hypertension may indicate that this FM cohort had increased prevalence of MetS. Moreover, we found that body composition variables (especially VAT, BMI, ASAT, and T-MFI) were significantly associated with blood pressures in FM. The clinical importance of these variables is emphasized by population-based data reporting that high VAT in combination with high or low liver fat are associated with atherosclerosis and increased risks for cardiovascular disease and type 2 diabetes.^21^ Resting blood pressure shows a negative relationship with pain sensitivity (blood pressure – related hypoalgesia) – ie, it is a pain inhibitory mechanism in healthy subjects and may involve endogenous opioids and α_2_-adrenergic pathways.^110^,^116–118^ In chronic pain conditions including FM dysfunction of this mechanism has been demonstrated.^110^,^116^,^117^ In subjects with resolved chronic pain, this mechanism is more effective than in patients with chronic pain.^116^ Obesity is associated with increased sensitivity to nociceptive stimuli in healthy subjects and in FM.^23^,^119^,^120^ In this study, FM had lower pain thresholds according to PPT (Table 1). However, we could not significantly regress PPT using BMI and other fat infiltration variables in FM, which could be due to the relatively small FM cohort.

## Strengths and Limitations

We used MVDA to determine the relative importance of variables for group membership and in relation to clinical variables. Another strength is that we assessed the major adipose tissue and ectopic fat compartments and could do a more detailed link to body composition than would be possible with measures such as BMI. One limitation is the cross-sectional design, which does not allow for causal conclusions. Another limitation is the sample size, and our results need to be confirmed in larger studies. The 1990 ACR criteria for FM was used to compare with earlier studies. In future studies, both the 1990 ACR and newer criteria should be used.

### Clinical Implications

The proportion of obesity was >6 times higher in FM. The patients also had smaller thigh muscles and higher MFI in both muscle groups. This study as well as other studies show that obesity has an additional negative influence on FM symptomatology.^39^,^121^ Our findings must be considered from the perspective that obesity is associated with increased risk for serious conditions such as type 2 diabetes, cardiovascular diseases, cancer, and dementia.^122^,^123^ Moreover, obesity can be considered a pain facilitator with contributions via mechanical stress, chronic low-grade inflammation, and neuroimmune factors in chronic pain including FM.^123^,^124^ Physical exercise can reverse part of obesity-related pathologies and result in lower body fat and weight,^19^ and there is clear evidence that FM patients benefit from physical exercise with respect to important clinical variables.^33^,^96^,^125^,^126^ Generally, nutrition interventions are effective on pain in patients with chronic pain conditions^127^ and are recommended by IASP. Hence, it is important to evaluate nutrition interventions in FM.^128^ Both physical exercise and a change in diet involve long-term behavioral changes and one should not underestimate the difficulties of making such changes when one has generalized pain along with frequent comorbidities, indeed several psychological factors could act as facilitators (eg, pain acceptance) or barriers (eg, pain catastrophizing),^42^ thus a multidisciplinary treatment is recommended.

## Conclusion

The visceral, subcutaneous, liver, and muscle fat infiltration variables and BMI were significantly higher in FM, whereas muscle volume was lower in quadriceps but not in spinal erectors. Physical tests indicate deconditioning in FM. Physical capacity variables and several of the fat infiltration variables correlated negatively in FM. Body composition variables and physical capacity variables were significantly associated with group belonging (FM or CON). Both pain intensity and FIQ were significantly associated with a blend of body composition and physical capacity variables. The physical capacity variables were generally more important for group, pain intensity, and FIQ than fat infiltration variables. However, in FM, blood pressures were strongest and significantly associated with the body composition variables. Clearly, considering obesity, dietary habits, and level of physical activity should become very important in the clinical management of patients with FM.

## Data Sharing Statement

The datasets generated and/or analyzed in this study are not publicly available as the Ethical Review Board has not approved the public availability of these data.
